# News and views

**DOI:** 10.1007/s43673-021-00013-4

**Published:** 2021-05-31

**Authors:** AAPPS

**Affiliations:** Association of Asia Pacific Physical Societies, Pohang, Korea

## Exploring antiquark asymmetry in protons by Wen-Chen Chang and Shin’ya Sawada

Protons and neutrons, the components of every nucleus, are the dominating mass carriers of our visible universe. Our understanding of protons and neutrons (i.e., nucleons) has been evolving from a simple 3-quark structure to the 1d longitudinal momentum fraction (*x*) distributions of quarks and gluons and the multi-dimensional distributions with additional dependency on transverse size or transverse momentum.

Inside the nucleons, there is the existence of anti-quarks, originated from the splitting of gluons, the strong force carrier. Utilizing the Drell-Yan process, the NuSea/E866 experiment identified a large asymmetry of anti-d and anti-u asymmetry over the range of *x*=0.02–0.35, with an intriguing signal of a “turning over” at large *x*. This turning-over trend is puzzling since it is not compatible with theoretical pictures such as the pion-cloud model, which can account for the observed large antiquark asymmetry.

**Fig. 1:**
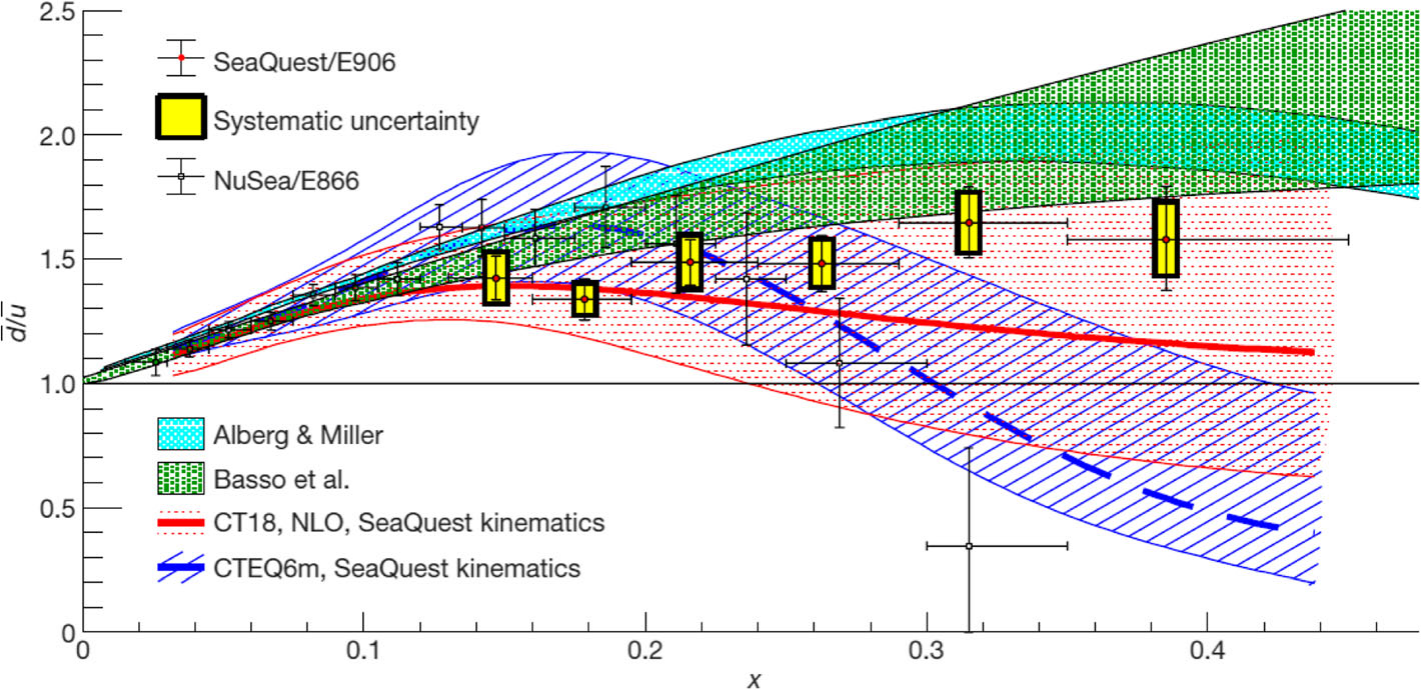
Ratios of $\overline{d}(x)$ to $\overline{u}$(*x*) in the proton (red filled circles) from the SeaQuest/E906 data, based on next-to-leading-order calculations of the Drell-Yan cross sections. Results of the previous measurement by the NuSea experiment, the calculated ratios with two PDFs, and the predictions of two theoretical models are shown for comparison. The figure is taken from *Nature* 590, 561 (2021)

In 2008, a joint team from the USA, Japan, and Taiwan began working on the Fermilab SeaQuest/E906 experiment, whose measuring capability was on the large-*x* region. Their results regarding large-*x* antiquark asymmetry have been published in *Nature* 590, 561 (2021) https://www.nature.com/articles/s41586-021-03282-z, along with commentary at https://www.nature.com/articles/d41586-021-00430-3. The results clearly show that these distributions of antiquarks are considerably different, with more abundant anti-d than anti-u over a wide range of *x*, up to 0.45. The measurements will improve the accuracy of the anti-quark distributions of protons and are important for the studies at the Large Hadron Collider at CERN in terms of establishing the standard model baseline in the search of new physics. In addition, the results should motivate further theoretical and experimental studies to understand nucleon structure, such as more sophisticated models and measurements in charactering the spin and mass decomposition of nucleons (Fig. 1).

## Perovskite-quantum-dot plasmonic nanolasers with ultralow thresholds by Yu-Jung Lu

To date, the size of conventional lasers, which is usually larger than half of their wavelength due to the diffraction limit, hinders their applications in on-chip integration and ultracompact optoelectronic devices. The miniaturization of optoelectronic components has gradually become an important research topic, especially for nanolasers. How can the size of lasers be reduced? How can the power consumption of laser operations be reduced? How can a laser with a new working mechanism be developed to overcome the diffraction limit? These questions have become the key challenges for nanolaser research. Recently, Prof. Yu-Jung Lu, an assistant research fellow at the Research Center for Applied Sciences at Academia Sinica, and her collaborative research teams have published an article on continuous-wave nanolasing from an inorganic lead-halide perovskite (CsPbBr_3_) quantum dot in a gap-plasmon nanocavity with an ultralow threshold at 120 K. This research provides an approach for realizing on-chip electrically driven lasing and integration into plasmonic circuitry for information processing. This work was published in *ACS Nano* as a selected cover issue in 2020.

**Fig. 2:**
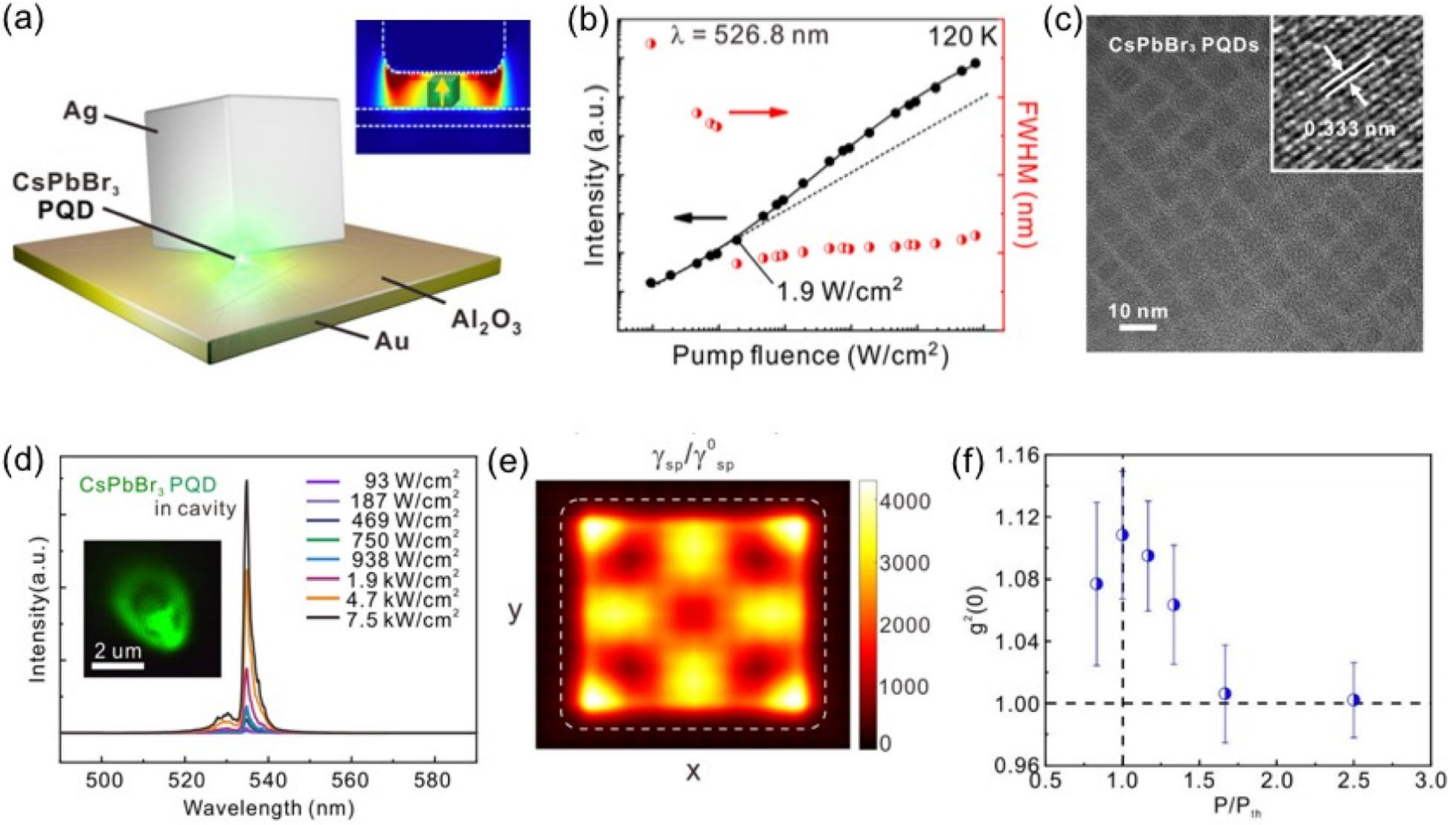
**a**-**e** Schematic, lasing signatures, and the lasing mechanism of a single perovskite quantum dot (PQD) in a gap plasmon nanocavity at 120 K. **f** The temporal coherence signature of the PQD nanolasing under 120 K was determined

The nanocavity is designed as a sandwich structure in which a single highly emissive perovskite quantum dot is located between a silver nanocube and a gold substrate, as shown in Fig. 2a. A thin layer of Al_2_O_3_ was deposited between the perovskite quantum dot and the gold substrate to avoid PL quenching. This was the first continuous wave operation of a perovskite nanolaser at a wavelength of 534 nm with an ultralow lasing threshold of 1.9 W cm^−2^ at 120 K (see Fig. 2b, d), and it had a temporal coherence feature for measuring the second-order correlation function (see Fig. 2f). More significantly, it set a state-of-the-art record for the ultrasmall localized mode volume (~0.002 λ^3^), which was two orders of magnitude smaller than the optical diffraction limit.

A key reason for this success was the development of perovskite quantum dots, which were prepared by a novel spray synthesis method that has extraordinary optical properties. A transmission electron microscopy image of the synthesized CsPbBr_3_ perovskite quantum dots is shown in Fig. 2c. Note that a semiconductor with high crystallinity may eliminate the nonradiative recombination pathways and enhance the emission. Thus, the features of high quantum yield and high optical gain coefficient make the device easily achieve lasing. The lead author, Yu-Hung Hsieh, who is a PhD student at National Tsing-Hua University, theoretically shows that the localized Purcell enhancement and the coupling strength for the single quantum emitter can be tailored by varying the dipole orientation and the position of the quantum dot (see Fig. 2a and e). Under particular conditions, lasing can be achieved. The ultrasmall mode volume of the nanolaser leads to desirable device properties, such as low-power and fast-switching, which will be beneficial in applications in future ultracompact integrated circuits for optical communication and information processing [1].

## AAPPS-DACG Workshop by Misao Sasaki and Sang Pyo Kim

The executive committee (EXCO) of the Division of Astrophysics, Cosmology and Gravitation (DACG) decided to organize a DACG Workshop on astrophysics, cosmology and gravitation, given that the global pandemic of coronavirus forced the Cosmology and Particle Astrophysics (CosPA) Symposium 2020, sponsored by DACG, to be postponed to November 2021. Many researchers in the Asia Pacific region have been relatively isolated from outside universities or institutions, and the International Organizing Committee of all members of the EXCO and the Local Organizing Committee (Kiwoon Choi, Bogeun Gwak, Gungwon Kang, Sang Pyo Kim, Sungwon Kim) of the DACG Workshop anticipated that this event would encourage those researchers in the region to promote and disseminate their recent works, and to exchange ideas and communicate with each other.

**Fig. 3:**
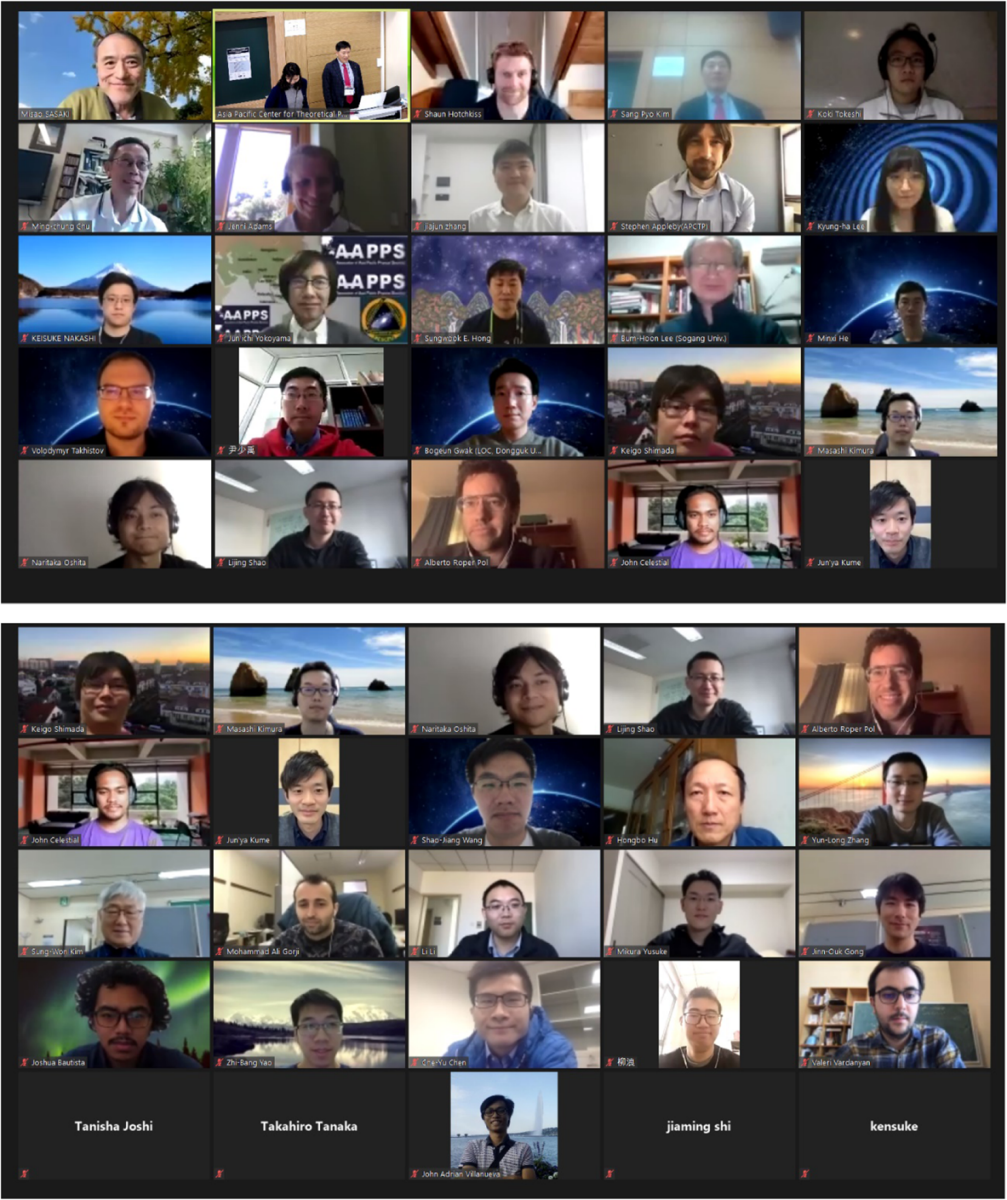
Photos taken during the 2020 DACG Workshop

The DACG Workshop was hosted by the Asia Pacific Center for Theoretical Physics (APCTP) as a hybrid online and onsite meeting from November 9 to 13, 2020. The workshop was attended by 213 participants. There were nineteen plenary speakers: Filipe B. Abdalla (University College London), Celine Boehm (Sydney University), Bernard Carr (Queen Mary University London), Chiang-Mei Chen (National Central University), Cheng-Wei Chiang (National Taiwan University), Ming-chung Chu (Chinese University of Hong Kong), Teruaki Enoto (Institute of Physical and Chemical Research (RIKEN)), Paul Ho (Institute of Astronomy & Astrophysics, Academia Sinica (ASIAA)), Hongbo Hu (Institute of High Energy Physics (IHEP), Chinese Academy of Sciences (CAS)), Shaun Hotchkiss (Auckland University), Kenta Hotokezaka (Tokyo University), Sang Hui Im (Institute for Basic Science (IBS)-Center for Theoretical Physics of the Universe (CTPU)), Aya Ishihara (Chiba University), Hyung Mok Lee (Korea Astronomy and Space Science Institute (KASI), Seoul National University), Sherry Suyu (Max Planck Institute, Technical University Munich), Kent Yagi (University of Virginia), Masahito Yamazaki (Kavli Institute for the Physics and Mathematics of the Universe (Kavli IPMU)), Shuang-Nan Zhang (IHEP, CAS), and Zong-Hong Zhu (Beijing Normal University). There were 53 oral presentations (Fig. 3).

**Fig. 4:**
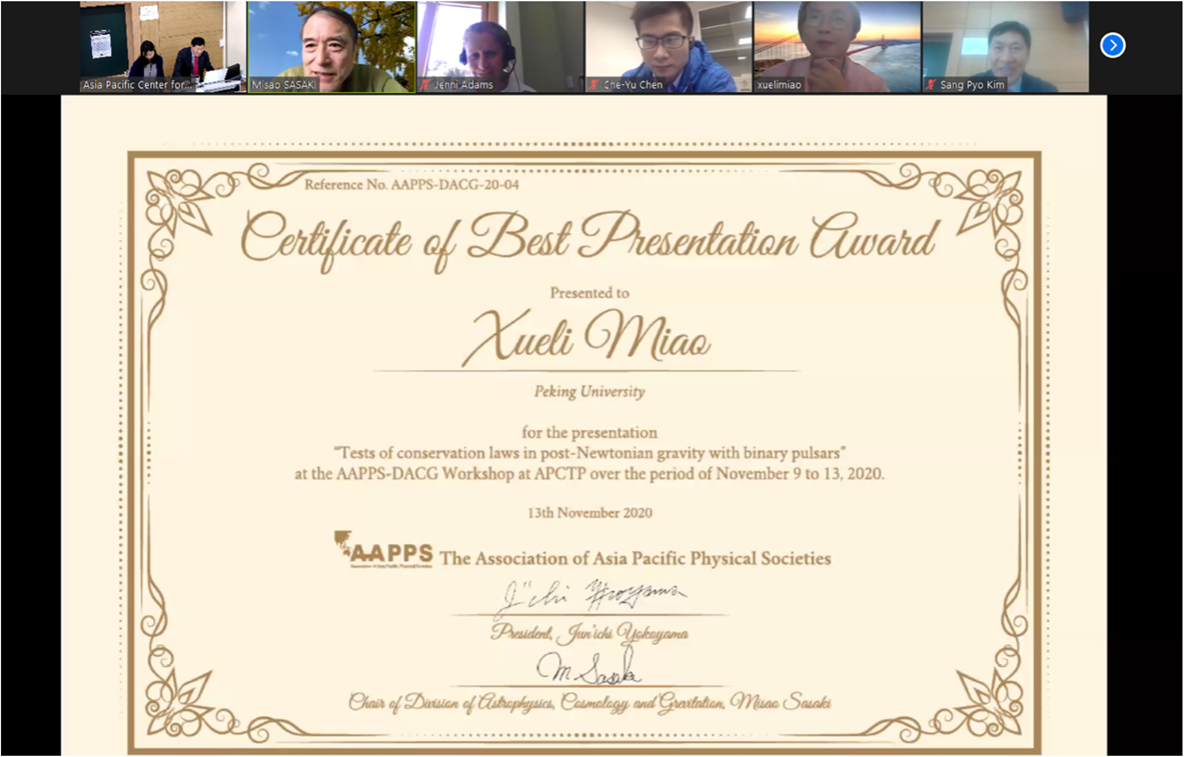
The Certificate of the Best Presentation Award for Xueli Miao, a graduate student

**Fig. 5:**
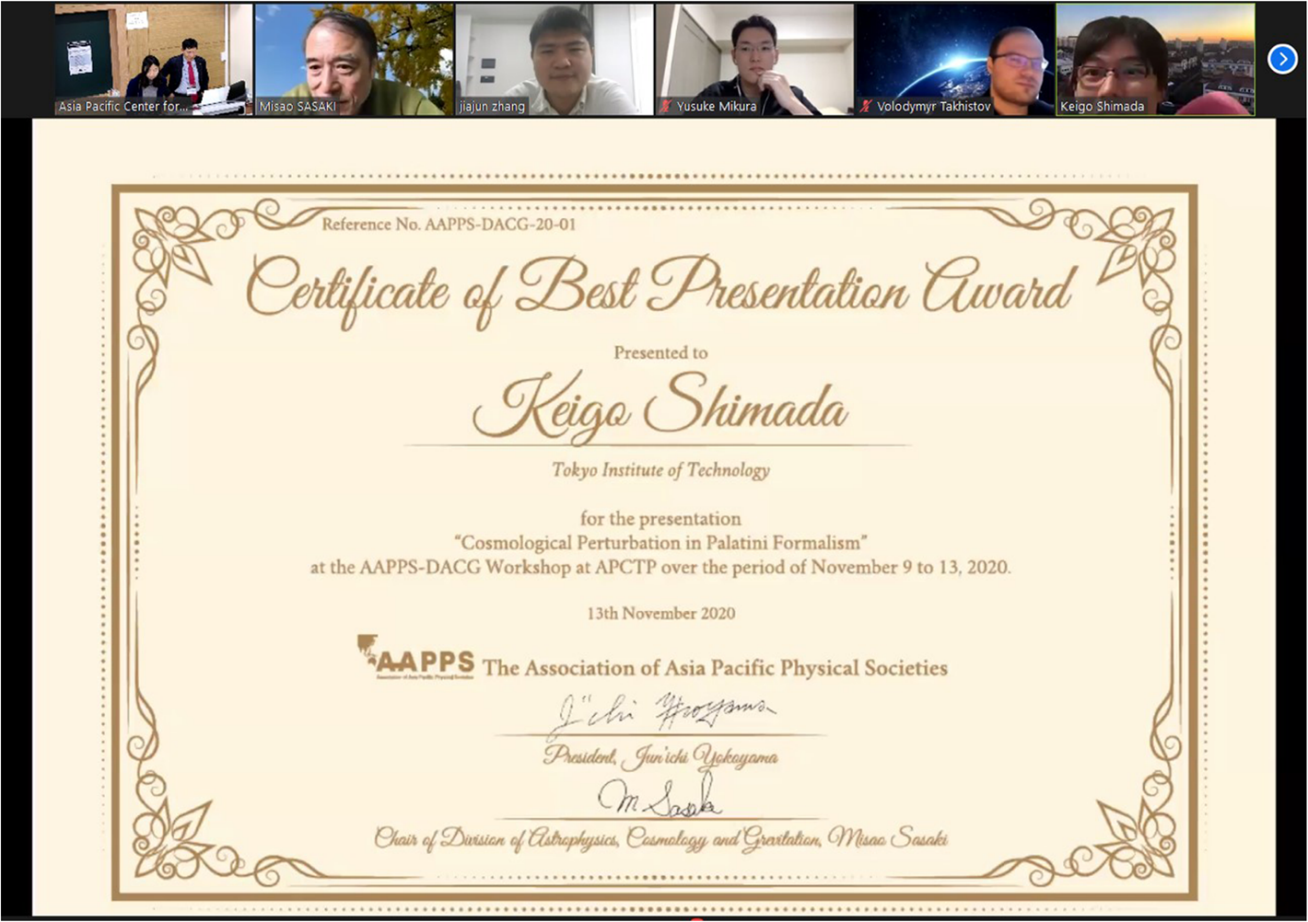
The Certificate of the Best Presentation Award for Keigo Shimada, a graduate student

The DACG together, with the Asia Pacific Cosmology and Particle Astrophysics Organization (AP CosPA Org.), awarded excellent presentations by graduate students and postdoctoral researchers, for whom not more than 6 years had passed since the completion of their respective PhDs. The awards will include full support of local expenses at CosPA 2021 in Hong Kong, which will be endorsed by DACG. The program committee selected the seven awardees: Che-Yu Chen (Institute of Physics, Academia Sinica), Chan Park (Korea Institute of Science and Technology Information (KISTI)), Volodymyr Takhistov (Kavli IPMU, Tokyo University), Xueli Miao (Peking University), Jiajun Zhang (IBS-CTPU), Yusuke Mikura (Nagoya University), and Keigo Shimada (Tokyo Institute of Technology) (Figs. 4 and 5).

The DACG Workshop was followed by another workshop, Astroparticle: the next frontier of fundamental physics, which was organized by Stephen Appleby and Eoin Colgain of APCTP from November 16 to 19, 2020. This workshop brought together experts in the fields of astronomy and particle physics. The 24 invited speakers gave talks from the standard model of particle physics to cosmology.

## The Physical Society of Japan announces the recipients of the 26th Outstanding Paper Award

In recognition of important achievements toward progress in physics, the Physical Society of Japan (JPS) annually selects outstanding papers from among original research articles published in the *Journal of the Physical Society of Japan*, *Progress of Theoretical Physics*, *Progress of Theoretical and Experimental Physics*, and *JPS Conference Proceedings*. The selection committee has chosen five papers for the 2021 award based on 19 nominations (for 16 papers) made by the editors of the JPS journals and representatives of the 19 divisions of the JPS.

On the morning of March 14, 2021, the 2021 award ceremony was held as a Zoom webinar.

The titles of the five selected papers, together with their citations, follow below.

1. Observation of a Be double-Lambda hypernucleus in the J-PARC E07 experiment **Prog. Theor. Exp. Phys. 2019, 021D02 (2019) H Ekawa, K Agari, J K Ahn, T Akaishi, Y Akazawa, S Ashikaga, B Bassalleck, S Bleser, Y Endo, Y Fujikawa, N Fujioka, M Fujita, R Goto, Y Han, S Hasegawa, T Hashimoto, S H Hayakawa, T Hayakawa, E Hayata, K Hicks, E Hirose, M Hirose, R Honda, K Hoshino, S Hoshino, K Hosomi, S H Hwang, Y Ichikawa, M Ichikawa, M Ieiri, K Imai, K Inaba, Y Ishikawa, A Iskendir, H Ito, K Ito, W S Jung, S Kanatsuki, H Kanauchi, A Kasagi, T Kawai, M H Kim, S H Kim, S Kinbara, R Kiuchi, H Kobayashi, K Kobayashi, T Koike, A Koshikawa, J Y Lee, J W Lee, T L Ma, S Y Matsumoto, M Minakawa, K Miwa, A T Moe, T J Moon, M Moritsu, Y Nagase, Y Nakada, M Nakagawa, D Nakashima, K Nakazawa, T Nanamura, M Naruki, A N L Nyaw, Y Ogura, M Ohashi, K Oue, S Ozawa, J Pochodzalla, S Y Ryu, H Sako, Y Sasaki, S Sato, Y Sato, F Schupp, K Shirotori, M M Soe, M K Soe, J Y Sohn, H Sugimura, K N Suzuki, H Takahashi, T Takahashi, Y Takahashi, T Takeda, H Tamura, K Tanida, A M M Theint, K T Tint, Y Toyama, M Ukai, E Umezaki, T Watabe, K Watanabe, T O Yamamoto, S B Yang, C S Yoon, J Yoshida, M Yoshimoto, D H Zhang, Z Zhang**

The lambda particle is the lightest hyperon that contains one strange quark. In order to determine the structure of neutron stars, one of the most important pieces of information is the interaction between lambda particles. This interaction plays a major role in gravitational waves generated from the merger of neutron stars and in r-process nucleosynthesis. Because the lambda particle is short-lived, studying the structure of double lambda hypernuclei is critical to determining the interaction between two lambda particles. This paper reported on the successful production of a double hypernucleus of ^11^_ΛΛ_Be. The experiment was performed at J-PARC by combining an emulsion technique and high-resolution spectrometry of charged particles. Even though seven events had previously been reported for the production of double hypernuclei, the nuclide of the produced hypernuclei had been uniquely identified in only one case. The present study constitutes the second case in which nuclides were successfully identified. The experimental data had much higher statistics than in previous measurements, and the _ΛΛ_ binding energy ΔB_ΛΛ_ could be determined with higher precision. The data confirmed that the attraction between L particles was significantly weaker than that between nucleons. Moreover, it was determined that the value of ΔB_ΛΛ_ of ^11^_ΛΛ_Be was significantly different from that of ^6^_ΛΛ_He, indicating that the value of ΔB_ΛΛ_ depended on the core nucleus in a hypernucleus. These results significantly improve our understanding of hyperon–hyperon interactions. It was therefore determined that this article deserves the Outstanding Paper Award of the Physical Society of Japan.

2. Superconductivity in Ca_1-x_La_x_FeAs_2_: A Novel 112-Type Iron Pnictide with Arsenic Zigzag Bonds **J. Phys. Soc. Jpn. 82, 123702 (2013) Naoyuki Katayama, Kazutaka Kudo, Seiichiro Onari, Tasuku Mizukami, Kento Sugawara, Yuki Sugiyama, Yutaka Kitahama, Keita Iba, Kazunori Fujimura, Naoki Nishimoto, Minoru Nohara, and Hiroshi Sawa**

Following the discovery of iron-pnictide superconductors (*T*_c_~26 K) by Hosono’s group, tremendous efforts have been devoted to synthesizing new superconductors with similar crystal structures and higher superconducting transition temperatures (*T*_c_). The highest *T*_c_ in this class of materials has increased to 55 K. The following research aimed at increasing *T*_c_ and developing new structures to investigate the relationship between the superconductivity mechanism and electronic dimensionality. For this latter purpose, various spacer layers between FeAs layers were developed.

This paper reported on the discovery of a new iron-based superconductor with a zigzag As chain as a spacer layer. Its distinct properties are described as follows: (i) the crystal structure is monoclinic, in contrast to most of the iron-based superconductors, which have an orthorhombic structure; (ii) the spacer layer is a one-dimensional zigzag chain of the As dimer; and (iii) although a sharp transition was observed at 34 K, the presented data indicated the possibility of a higher *T*_c_ of 45 K, which may be realized by adjusting the compositions.

Although signs of superconductivity in the Ca-La-Fe-As system were already known since the early stages of research on iron-based superconductors, its composition and structure have remained unknown. The present study provided a clear solution to this problem by synthesizing a theoretically proposed compound. This paper qualifies as a high-level experimental study and distinguishes itself from many other works reporting on new superconductors in the following points: the growth of high-quality single crystals, the determination of the electron density distribution map by maximum entropy method (MEM) analysis using X-ray diffraction data taken at SPring-8, and the determination of the Fermi surface by first-principles calculation.

Motivated by this study, many researchers have investigated this system and have actually reported a higher *T*_c_ (> 40 K) than that suggested in this study. This paper has contributed significantly to the study of iron-based superconductors, which is evidenced by the more than 100 citations. This work is a good example of high level Japanese research in the field of iron-based high-temperature superconductors, and therefore, this paper deserves the Outstanding Paper Award from the Physical Society of Japan.

3. Superconductivity of Au–Ge–Yb Approximants with Tsai-type Clusters **J. Phys. Soc. Jpn. 84, 023705 (2015) Kazuhiko Deguchi, Mika Nakayama, Shuya Matsukawa, Keiichiro Imura, Katsumasa Tanaka, Tsutomu Ishimasa, and Noriaki K. Sato**

The development of new superconductors and the elucidation of their non-trivial superconducting mechanisms have been vigorously studied globally. In particular, the following questions related to this area of research have been posed: “Does superconductivity appear in quasi crystals without crystal translational symmetry?” and “What is the mechanism of superconductivity?” Studies have been conducted to answer these perplexing questions.

In this paper, the authors reported on the discovery of superconductivity for two novel Tsai-type cluster compounds: AGY(I) [Au_64.0_Ge_22.0_Yb_14.0_, *T*_c_ = 0.68 K] and AGY(II) [Au_63.5_Ge_20.5_Yb_16.0_, *T*_c_ = 0.36 K]. These are called “approximant crystals” and are located between ordinary crystals and “quasicrystals”. In particular, in AGY(II), structural analysis has revealed that magnetic Yb ions occupy the center of the Tsai cluster, and the superconducting mechanism has been investigated in terms of its correlation with magnetism.　Recently, the authors also discovered superconductivity (*T*_c_ = 0.05 K) in the “quasicrystal” Al-Zn-Mg [K. Kamiya, et al., Nat. Commun. 9, 154 (2018)], which is a development of this “approximant crystal,” and non-trivial superconductivity has been investigated. In this paper, the first superconductivity in “approximant crystals,” which is the cornerstone of “quasicrystals” showing superconductivity, is reported, and the mechanism is described as a curious superconductivity involving magnetic ions, which is a discovery that has tremendous impact. For these reasons, we determined that the present paper deserves the Outstanding Paper Award of the Physical Society of Japan.

4. Anomaly Polynomial of General 6D SCFTs **Prog. Theor. Exp. Phys. 2014, 103B07 (2014) Kantaro Ohmori, Hiroyuki Shimizu, Yuji Tachikawa, Kazuya Yonekura**

In this study, the authors developed a method to obtain anomaly polynomials systematically in general six-dimensional *N* = (2,0) and *N* = (1,0) superconformal field theories (SCFT), and they demonstrated this in many concrete examples. The study of this type of six-dimensional SCFT is important in both string theory and quantum field theory. It often appears in string theory, as it is realized on a six-dimensional object called an M5-brane in M-theory, which is considered to be a unified theory of string theory. Furthermore, it has remarkable properties with profound applications. For example, dualities in some four-dimensional gauge theories can be derived by compactifying this six-dimensional theory to four dimensions. However, in general, analysis of this theory is extremely difficult, as no reliable weakly coupled description exists. Even the Lagrangian has not been written down in a covariant form because it contains a rank-2 antisymmetric tensor field with a self-dual field strength. The major achievement of this study is that it succeeds in determining the structure of an anomaly by circumventing all these difficulties.

The authors noticed that even if the scalar field related to the antisymmetric tensor field by supersymmetry acquired a non-zero vacuum expectation value, the symmetry of interest was not broken and the anomaly remained unchanged. Based on this fact, an analysis was performed in an appropriate vacuum in which the low-energy effective theory became simpler. In this case, the contribution of the anomaly from the one loop diagram was already known, and what they needed to evaluate was the contribution of the so-called GS term found by Green and Schwarz, which may exist in a theory with antisymmetric tensor fields. They showed that the GS term could be determined in quite general cases by imposing gauge anomaly cancellation and by comparing the results with compactified theories. Applying this idea, they successfully obtained explicit expressions for various systems, reproduced known results, and provided new formulas.

They also made an interesting observation, namely, that the number of physical degrees of freedom suggested by the anomaly analysis was proportional to *Q*^3^, where *Q* is the number of M5-branes in the large *Q* limit. This was consistent with the prediction of the AdS_7_/CFT_6_ correspondence in M-theory. They also confirmed that their results were consistent with the anomaly inflow in M-theory.

This paper provided reliable and fulfilling insights and has had tremendous influence as a basic study in this field. Therefore, we determined that this paper deserves the Outstanding Paper Award of the Physical Society of Japan.

5. Quantum Thermal Hall Effect in a Time-Reversal-Symmetry-Broken Topological Superconductor in Two Dimensions: approach from Bulk Calculations **J. Phys. Soc. Jpn. 82, 023602 (2013) Hiroaki Sumiyoshi and Satoshi Fujimoto**

This study examined the thermal Hall conductivity (THC) of two-dimensional topological superconductors. The authors derived the formula of THC from the Bogoliubov–de Gennes (BdG) equation and showed that its coefficient is given by half of the Chern number, that is, it is quantized to one-half of that in the quantum Hall system. This half quantization itself was predicted by other authors based on the picture of the Majorana edge state. The present work is original in the derivation from the BdG equation that describes bulk states and in expressing the result using the bulk topological (Chern) number. The formula was derived without limiting the generality and is quite versatile, e.g., it is not restricted to two dimensions. In fact, the formula has been applied to three-dimensional chiral superconductors with a nodal gap [N. Yoshioka et al., J. Phys. Soc. Jpn. **87**, 124602 (2018)]. As observed experimentally in a Kitaev magnet α-RuCl^3^, [Y. Kasahara et al., Nature **559**, 227 (2018)] half quantization of THC is currently a hot topic in the field of topological materials, to which this study made a significant contribution.

In their calculation, the authors used Luttinger’s gravitational potential and followed the recent leading-edge transport theory that considers energy magnetization along with the Kubo formula [T. Qin et al., Phys. Rev. Lett. **107**, 236601 (2011)]. Examining carefully the crucial role in the half quantization played by the particle-hole symmetry of the BdG equation, the present work extended the aforementioned transport theory for normal metals and insulators to superconductors.

These discoveries show that this paper deserves the Outstanding Paper Award of the Physical Society of Japan.

## The inaugural message from the newly elected president of KPS by Tae-Won Noh

**Fig. 6:**
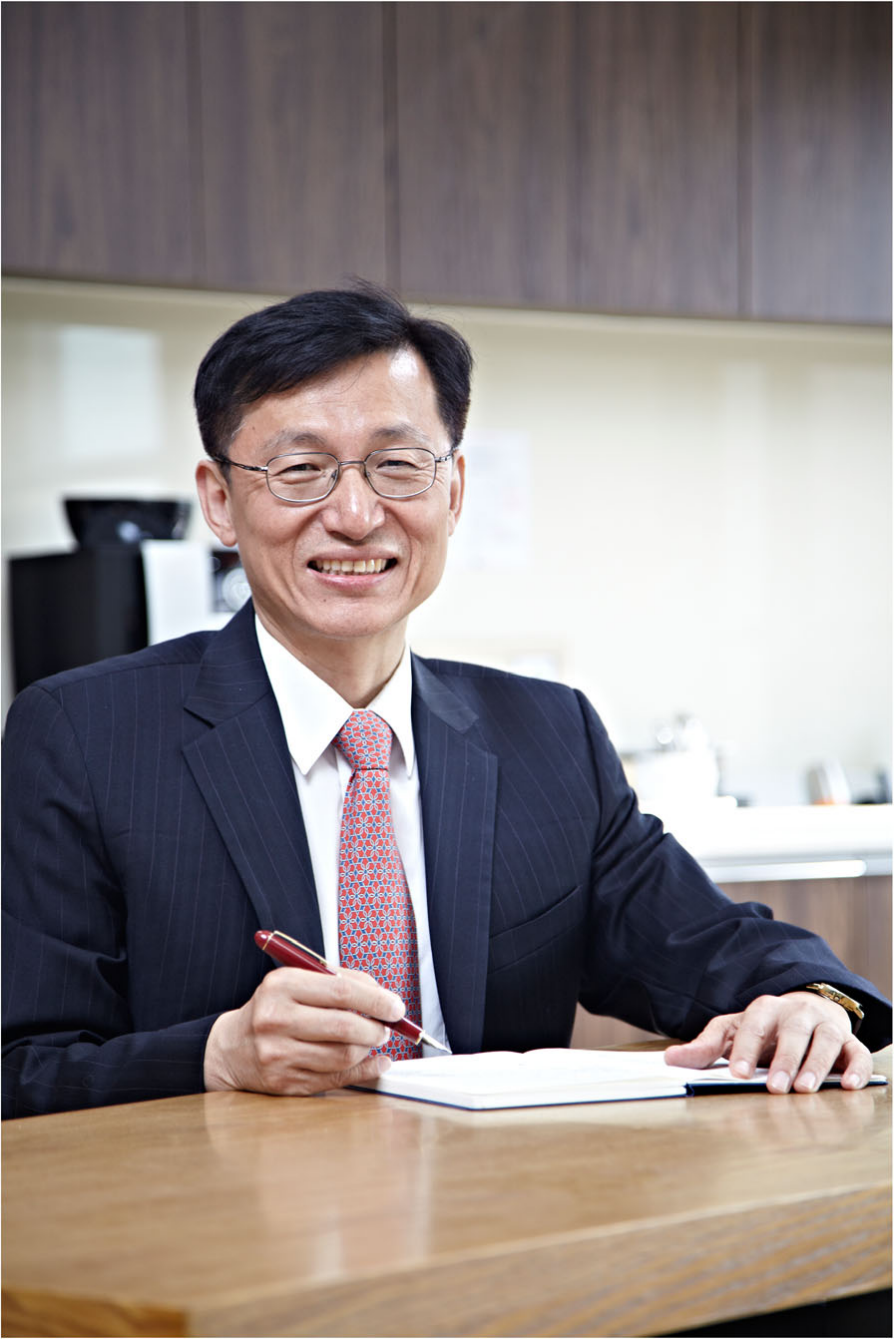
The newly elected President Tae-Won Noh

 As of January 1, 2021, Professor Tae Won Noh of Seoul National University began his 2-year term as the 29th president of the Korean Physical Society (KPS) Fig 6. The 29th executive body of the KPS is composed of, among others, the executive vice-president, Professor Intae Yu (Sungkyunkwan University); the secretary of general affairs, Professor Takhee Lee (Seoul National University); and the treasurer, Professor Baeho Park (Konkuk University). At the first council meeting on January 8, 2021, President Noh gave the following inaugural message for KPS members.

First of all, I hope that all the members of KPS are in good health and are doing well. As is well known, most members are experiencing many difficulties due to the unexpected COVID-19 pandemic. KPS also suffered from unprecedented difficulties as the annual spring KPS conference had to be postponed, although it was later switched to an online conference. Now, KPS must face the era of the so-called “new-normal”. The members of KPS should play a pivotal role in overcoming the current challenges by being part of the transformation of KPS.

Since its founding in 1952, KPS has undergone prominent growth through the dedication of its members. Unfortunately, we are now facing unparalleled challenges. The gap between the rich and the poor universities is getting bigger while the ecosystem of physics education is being threatened by the decline of the school-age population as well as the reduction of physics education in middle and high schools. To overcome this painful reality, members should play central role in being part of a bold and creative transformation. Based on my experiences so far, I would like to lay stepping stones for this transformation, together with you.

First, we will significantly improve the operation of on-line conferences, especially given the trend to conduct conferences remotely. By introducing an on-off hybrid system suitable for latest “new-normal” era, new changes in the operation of conferences will be implemented. To this end, we will make a bold investment in restructuring our system and organization for conferences, which includes constructing the requisite infrastructure for these ends.

Second, we will activate new physics education and business programs that can meet the needs of the times. Now is the time to introduce new physics programs that can meet modern demands, such as quantum computers and artificial intelligence. We will come up with a variety of measures to make the necessity and importance of physics become clear not only to those in the science and engineering communities, but also to our society as a whole.

Third, we will be reestablished as an academic society that is of practical help to members. Through the allocation of research funds via “block funding”, we will use our influence for local universities and small academic fields. We will do our best to prevent disruption in careers for successive generations of academics and strengthen the activities of local branch and academic journal services.

Fourth, to shape a desirable ecosystem for physics community, we will encourage various members to participate in our society. It is necessary to expand opportunities for women who are members to participate in conferences, and to create a system for sharing and cooperating roles that fit the characteristics of universities, research institutions, and industries. Long-term efforts are necessary for changes in the ecosystem, and essential organizations will be installed and activated.

Now we must work to create a win-win situation through dialogue and cooperation. I will continuously visit members so that I can become a channel of communication by hearing opinions from various members. The Korean Physical Society will do its best to undergo this transformation and to make a new leap forward. I thank you, the members of the Korean Physical Society, for your interest and encouragement.

## The Australian Institute of Physics elects a new executive by Kirrily Rule

In February 2021, the Australian Institute of Physics elected their new National Executive team by unanimous vote. Stepping into the role of President of the AIP is Prof. Sven Rogge who is also the Pro Vice-Chancellor (Research) at the University of New South Wales, Sydney (UNSW). Sven’s research interest is in condensed matter physics, in particular quantum electronics, at the School of Physics. Sven works on quantum computation in silicon in the ARC Centre for Quantum Computation and Communication Technology. Sven takes over this role from Professor Jodie Bradby from ANU who is now advising the Executive as the Immediate Past President. Following her 2-year role as President of the AIP, Jodie is now dedicating her time as the Physical Sciences Representative for Science and Technology Australia.

New to the Executive committee this year is Prof. Nicole Bell from the University of Melbourne who joins as our Vice President. Nicole’s physics research interests lie at the intersection of Particle Physics, Astrophysics and Cosmology. Nicole is also the Theory Program Leader for the ARC Centre of Excellence for Dark Matter Particle Physics.

Another new member to join the committee this year is Dr. Joanna Turner from the University of Southern Queensland who has taken on the role of Special Projects Officer for awards. Part of Joanna’s role in the executive will be to coordinate the awards and prizes that are presented each year by the AIP. Joanna has also been key in reviewing the current awards and processes for nomination, adjudication, and presentation of these awards.

Returning to their roles as Honorary Treasurer, Honorary Registrar, and Honorary Secretary are Dr. Judith Pollard, Prof. Stephen Collins, and Associate Professor Kirrily Rule, respectively. Associate Professor Dr *Gerd* Schröder-*Turk also returns in his capacity as the Special Projects Officer for Policy.* In addition, two additional Special Projects Officer roles have been formed to complement the Executive team. Dr. Stephan Rachel holds the Special Project Officer role for Finance while Dr. Timothy van der Laan is the Special Project Officer for Outreach.

**Fig. 7:**
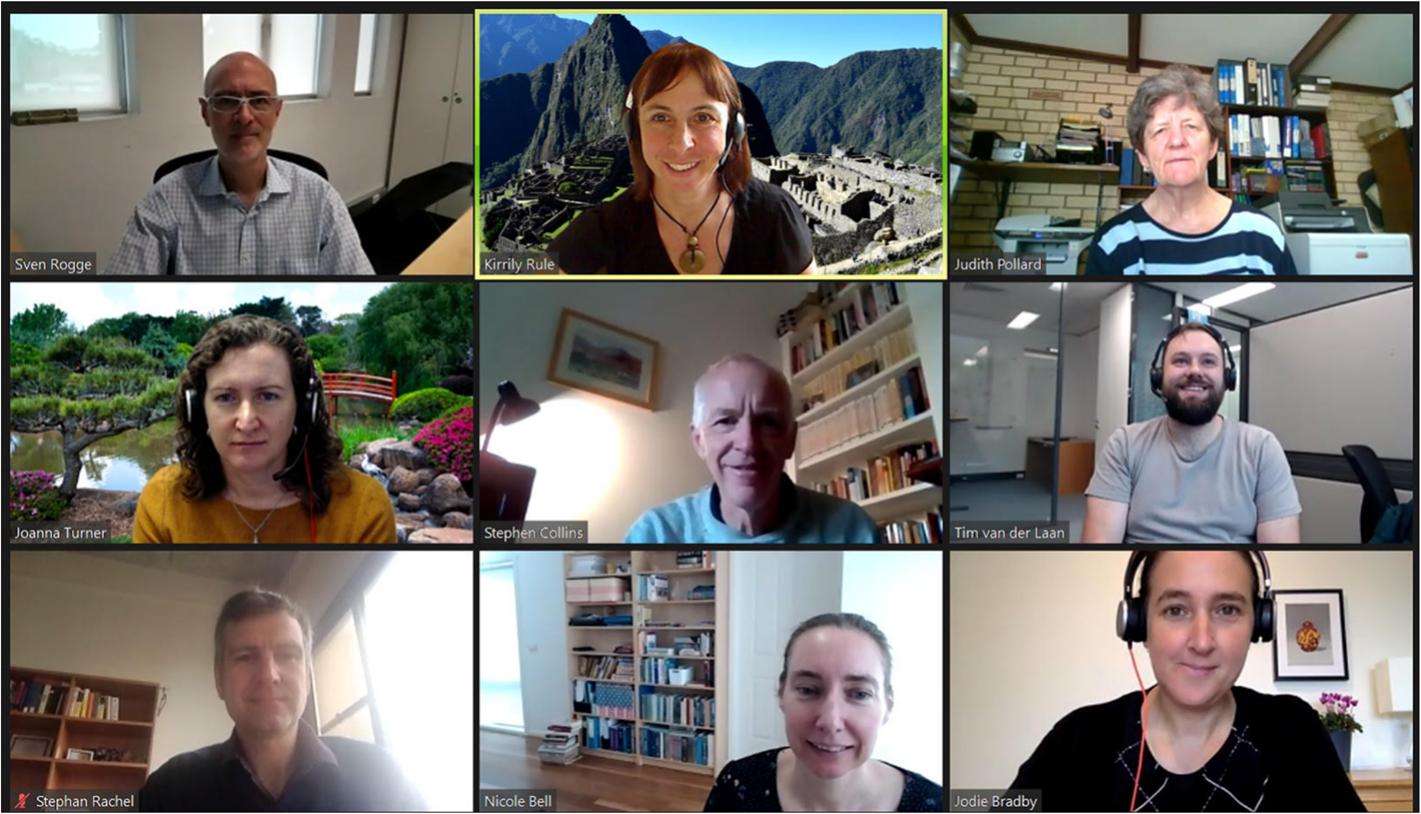
Some of the new faces of the AIP executive team during a recent Zoom meeting

This year’s Executive team has embraced diversity with members coming from 6 of the 7 states and territories that the AIP represents, indicating a strong National balance. Also notable is the perfect 50:50 split of women and men on the team (Fig. 7). We look forward to the next 2 years representing the Australian Physics community and promoting the role of Physics in research, education, industry, and the community.
